# State- and Condition-Dependent Modulation of the Hindlimb Locomotor Pattern in Intact and Spinal Cats Across Speeds

**DOI:** 10.3389/fnsys.2022.814028

**Published:** 2022-02-09

**Authors:** Jonathan Harnie, Johannie Audet, Stephen Mari, Charly G. Lecomte, Angèle N. Merlet, Gabriel Genois, Ilya A. Rybak, Boris I. Prilutsky, Alain Frigon

**Affiliations:** ^1^Department of Pharmacology-Physiology, Faculty of Medicine and Health Sciences, Centre de Recherche du CHUS, Université de Sherbrooke, Sherbrooke, QC, Canada; ^2^Department of Neurobiology and Anatomy, College of Medicine, Drexel University, Philadelphia, PA, United States; ^3^School of Biological Sciences, Georgia Institute of Technology, Atlanta, GA, United States

**Keywords:** locomotion, spinal transection, sensory feedback, central pattern generator, speed

## Abstract

Locomotion after complete spinal cord injury (spinal transection) in animal models is usually evaluated in a hindlimb-only condition with the forelimbs suspended or placed on a stationary platform and compared with quadrupedal locomotion in the intact state. However, because of the quadrupedal nature of movement in these animals, the forelimbs play an important role in modulating the hindlimb pattern. This raises the question: whether changes in the hindlimb pattern after spinal transection are due to the state of the system (intact versus spinal) or because the locomotion is hindlimb-only. We collected kinematic and electromyographic data during locomotion at seven treadmill speeds before and after spinal transection in nine adult cats during quadrupedal and hindlimb-only locomotion in the intact state and hindlimb-only locomotion in the spinal state. We attribute some changes in the hindlimb pattern to the spinal state, such as convergence in stance and swing durations at high speed, improper coordination of ankle and hip joints, a switch in the timing of knee flexor and hip flexor bursts, modulation of burst durations with speed, and incidence of bi-phasic bursts in some muscles. Alternatively, some changes relate to the hindlimb-only nature of the locomotion, such as paw placement relative to the hip at contact, magnitude of knee and ankle yield, burst durations of some muscles and their timing. Overall, we show greater similarity in spatiotemporal and EMG variables between the two hindlimb-only conditions, suggesting that the more appropriate pre-spinal control is hindlimb-only rather than quadrupedal locomotion.

## Introduction

Locomotion in quadrupeds normally involves all four limbs performing coordinated movements to achieve a smooth forward progression while maintaining dynamic balance (Frigon, 2017). Spinal animals (i.e., animals with a spinal transection or spinalization) have been instrumental in our understanding of the neural control of locomotion, particularly its spinal control, by comparing the locomotor pattern in the intact and spinal states (Rossignol et al., 2006; Frigon, 2020). Spinal animals with a thoracic transection recover hindlimb locomotion due to the presence of spinal locomotor networks, called central pattern generators (**CPGs**), that interact with somatosensory feedback from the limbs (Rossignol et al., 2006; McCrea and Rybak, 2008; Rossignol and Frigon, 2011; Kiehn, 2016; Frigon et al., 2021). However, in spinal animals, the forelimbs are often placed on a stationary platform or suspended in the air while the hindlimbs perform locomotor movements (Forssberg et al., 1980b; Smith et al., 1982; Giuliani and Smith, 1985; Robinson and Goldberger, 1986; Barbeau and Rossignol, 1987; Lovely et al., 1990; Leblond et al., 2003). The spinal transection changes the neural control of locomotion, as pathways and structures within the central nervous system normally communicate with the spinal locomotor CPGs controlling each limb during quadrupedal locomotion (Grillner, 1981; Drew et al., 1996; Frigon, 2017). Moreover, placing the forelimbs on a stationary platform or suspending them in the air also changes the biomechanics; for example, by shifting more weight on the hindlimbs and elevating the angle of the trunk relative to the horizontal. In turn, these biomechanical changes influence the neural control.

Despite these neuromechanical differences, the pattern of hindlimb locomotion in spinal animals is almost always compared with intact quadrupedal locomotion. There are several differences between the hindlimb locomotor pattern obtained in the intact quadrupedal and spinal hindlimb-only states. For example, at a given speed, the cycle duration is shorter after spinalization, attributable primarily to a decrease in the support phase (in cats: Bélanger et al., 1996; de Leon et al., 1998; Frigon and Rossignol, 2008, in rats: Alluin et al., 2015). Stride and step lengths are also reduced after spinalization (in cats Bélanger et al., 1996; Frigon, 2012, in rats: Alluin et al., 2015). To maintain the same speed, spinal animals must step at a higher cadence. Although the muscle activity (EMG, electromyography) pattern is similar (flexor-extensor alternation, left-right alternation) before and after spinalization, there are some differences. The burst duration of muscles with a primarily flexor or extensor activation is generally decreased after spinalization while mean amplitude increases for flexor muscles and decreases for extensor muscles (in cats: Bélanger et al., 1996; Frigon and Rossignol, 2008; Rossignol et al., 2014).

Are these differences due to the state of the system (intact versus spinal) or because the forelimbs do not participate in locomotion, which affects both the neural control and the biomechanics. There was one previous study that compared hindlimb EMG activity during quadrupedal and hindlimb-only locomotion in intact cats and during hindlimb-only locomotion after spinal transection at a single moderate treadmill speed of 0.4 m/s (de Leon et al., 1998). The authors of this study found some differences in EMG activity between the three types of locomotion. Most notably they observed a double burst in the iliopsoas, a hip flexor, during hindlimb-only locomotion (intact and spinal) but not during quadrupedal intact locomotion. They also noted an earlier onset of the EMG activity of the tibialis anterior, an ankle flexor, during hindlimb-only locomotion (intact and spinal) compared to intact quadrupedal locomotion. Therefore, some changes in muscle activity were due to the type of locomotion (quadrupedal versus hindlimb-only) and not necessarily because of the change in the state of the system (intact versus spinal). However, several questions remain unanswered, as no analysis of spatiotemporal variables, such as stride/step lengths and cycle/phase durations or joint kinematics were made between the three locomotor conditions. Moreover, de Leon et al. (1998) did not specifically compare intact quadrupedal and hindlimb-only locomotion, focusing instead on comparisons with the spinal state.

Another question is how the intact cat adjusts the hindlimb locomotor pattern over a range of speeds during quadrupedal and hindlimb-only locomotion and how these adjustments differ during hindlimb-only locomotion in the spinal state. The main adjustments to speed in intact versus spinal cats during quadrupedal and hindlimb-only locomotion are the following: (1) the cycle, stance and extensor burst durations are reduced with increasing speed while swing phase and flexor burst durations remain relatively the same (Goslow et al., 1973; Halbertsma, 1983; Frigon et al., 2013; Dambreville et al., 2015; Harnie et al., 2018); (2) the step and stride lengths and the horizontal distance at liftoff between the toe and the hip increase with speed whereas the horizontal distance at contact remains relatively invariant (Bélanger et al., 1996; Frigon, 2012; Thibaudier and Frigon, 2014; Dambreville et al., 2015); (3) the mean EMG amplitude of flexor and extensor muscles increases with speed (Halbertsma, 1983; Pierotti et al., 1989; Frigon et al., 2014, 2015; Hurteau et al., 2017). What we do not know is if these adjustments in spinal cats more closely resemble those observed during intact quadrupedal or hindlimb-only locomotion. Comparing the three locomotor conditions could reveal state- and locomotor condition-dependent differences while providing a better basis for characterizing changes after spinal transection.

Therefore, the purpose of this study was to investigate the hindlimb locomotor pattern during quadrupedal and hindlimb-only locomotion in intact cats and in the same animals following spinal transection over a range of speeds. We hypothesized that the hindlimb locomotor pattern and its adjustment to speed in the spinal state more closely resemble the pattern obtained during hindlimb-only locomotion in the intact state.

## Materials and Methods

### Animals and Ethical Information

All procedures were approved by the Animal Care Committee of the Université de Sherbrooke and were in accordance with policies and directives of the Canadian Council on Animal Care (Protocol 442-18). Nine adult cats, 4 males and 5 females, weighing between 3.6 and 6.9 kg were used in the present study. We followed ARRIVE guidelines for animal studies (Percie du Sert et al., 2020). In our effort to reduce the number of animals used in research, we used these cats in other studies to answer different scientific questions (Latash et al., 2020; Harnie et al., 2019, 2021; Merlet et al., 2020, 2021).

### Surgical Procedures and Electrodes Implantation

We performed surgeries under aseptic conditions with sterilized instruments in an operating room. Before surgery, butorphanol (0.4 mg/kg), acepromazine (0.1 mg/kg), and glycopyrrolate (0.01 mg/kg) were injected intramuscularly for sedation and ketamine/diazepam (0.05 ml/kg, 1:1 ratio) for induction. Cats were anesthetized with isoflurane (1.5–3%) delivered in O_2_. Cats received a continuous infusion of lactated Ringers solution (3 ml/kg/h) during the surgery through a catheter placed in a cephalic vein. Anesthesia was maintained by adjusting isoflurane concentration as needed and by monitoring cardiac and respiratory rates. Body temperature was monitored with a rectal thermometer and maintained within physiological range (37 ± 0.5°C) using a water-filled heating pad placed under the animal and an infrared lamp ∼50 cm over it. We confirmed the depth of anesthesia by applying pressure to a paw (to detect limb withdrawal) and by assessing the size and reactivity of pupils. The animal’s skin was carefully shaved using electric clippers and cleaned with chlorhexidine soap.

We directed pairs of Teflon-insulated multistrain fine wires (AS633; Cooner Wire, Chatsworth, CA, United States) subcutaneously from two head-mounted 34-pin connectors (Omnetics Connector, Minneapolis, MN, United States). Electrodes were sewn into the belly of selected hindlimb muscles for bipolar recordings, with 1–2 mm of insulation stripped from each wire. The head connector was secured to the skull using dental acrylic. We verified electrode placement during surgery by electrically stimulating each muscle through the appropriate head connector channel.

At the end of surgery, we injected an antibiotic (Cefovecin, 0.1 ml/kg) subcutaneously and taped a transdermal fentanyl patch (25 mcg/h) to the back of the animal 2–3 cm rostral to the base of the tail for prolonged analgesia (4–5 day period). We also injected buprenorphine (0.01 mg/kg), a fast-acting analgesic, subcutaneously at the end of the surgery and ∼7 h later. After surgery, we placed the cats in an incubator until they regained consciousness. At the conclusion of the experiments, cats received a lethal dose of pentobarbital through the cephalic vein. To confirm that the spinal transection was complete in all cats, we performed histological analysis, which we visually presented in Harnie et al. (2019).

### Spinal Transection and Hindlimb Locomotor Recovery

For the spinal transection, general surgical procedures were the same as in the previous section. The skin was incised over the last thoracic vertebrae and after carefully setting aside muscle and connective tissue, a small dorsal laminectomy was made. After exposing the spinal cord, we applied xylocaine (Lidocaine hydrochloride, 2%) topically and made 2–3 intraspinal injections. We then completely transected the spinal cord with surgical scissors between the 12th and 13th thoracic vertebrae. The ∼0.5 cm gap between the two cut ends of the spinal cord was then cleaned and any residual bleeding was stopped. We verified that no spinal cord tissue remained connecting rostral and caudal ends. A hemostatic agent (Spongostan) was placed within the gap, and muscles and skin were sewn back to close the opening in anatomic layers. After spinal transection, we manually expressed the cat’s bladder and large intestine one to two times daily, or as needed.

As stated previously, seven cats were used in another study to describe the effects of three interventions on the recovery of standing and hindlimb locomotion after spinal transection (Harnie et al., 2019). In that study, cats were divided in three groups: two cats received manual therapy that consisted of distal to proximal strokes of the triceps surae muscles (0.33 Hz, 10 min per leg, 5 times a week for 5 weeks), two cats received locomotor training (20 min, 5 times a week for 5 weeks) that consisted of two experimenters moving the hindlimbs over the treadmill to reproduce locomotion, with one of the experimenters holding the tail for support, and three cats received no intervention. Cats recovered hindlimb locomotion without or with treadmill training, indicating that the recovery of hindlimb locomotion simply requires a return of excitability within spinal sensorimotor circuits. Based on this conclusion, the remaining two cats of the present study did not undergo any specific training intervention but were tested on a treadmill and during manual stimulation of the triceps surae muscles each week during recovery.

### Data Collection and Analysis

We collected data (EMG and kinematics) before (intact state) and after spinal transection (spinal state) during forward locomotion on a split-belt treadmill, with the left and right sides on separate belts. In the intact state, cats performed tied-belt (equal left-right speeds) locomotion in quadrupedal (Intact4) and hindlimb-only (Intact2) conditions. In the hindlimb-only conditions, the forelimbs were placed on a stationary platform. In the spinal state, we report data during hindlimb–only (Spinal2) locomotion only. During tied-belt locomotion, both sides stepped from 0.4 to 1.0 m/s in 0.1 m/s increments. We collected data from 6 to 15 consecutive step cycles in each locomotor condition. In the spinal state, we collected data with or without perineal stimulation, depending on the animal, to have a robust walking pattern. Data were collected between the sixth and ninth week after spinalization when cats were able to step on the treadmill up to a speed of 1.0 m/s. For perineal stimulation, an experimenter manually pinched the skin under the tail with the index finger and thumb. We did not provide weight support, although an experimenter gently held the tail to provide balance. To avoid fatigue, at least 20 s of rest were given between episodes of locomotion.

We collected kinematic data as described previously (Harnie et al., 2018, 2021). Videos of the left and right sides were obtained with two cameras (Basler AcA640-100g) at 60 frames/s with a spatial resolution of 640 by 480 pixels. A custom-made program (Labview) acquired the images and synchronized acquisition with EMG data. By visual detection, we determined limb contact as the first frame where the paw made visible contact with the treadmill surface, and limb liftoff as the most caudal displacement of the toe, for both hindlimbs. Cycle duration was measured from successive paw contacts, while stance duration corresponded to the interval of time from paw contact to liftoff. Swing duration was measured as cycle duration minus stance duration. Stride lengths were measured as the distance between stance onset and offset of a given limb added to the distance traveled by the treadmill during the swing phase, which was calculated by multiplying swing duration by treadmill speed (Courtine et al., 2005; Thibaudier and Frigon, 2014; Dambreville et al., 2015). Step lengths were measured as the distance between the leading and trailing limbs at stance onset of the leading limb (Hoogkamer et al., 2014). To reconstruct joint kinematics for the hindlimbs, we placed reflective markers over the iliac crest, greater trochanter, lateral malleolus, metatarsophalangeal joint and at the tip of the toes. The relative distance of the paw at contact and liftoff was measured as the horizontal distance between the hip and toe markers at stance onset and offset, respectively. The hip height was measured as the vertical distance between the hip and toe markers at stance onset. We also measured total angular excursion (Max – Min values) for the hip, knee and ankle joints across the cycle and the degree of yield at the knee and ankle (value at contact - value when switching from flexion to extension). We illustrated interjoint coordination using hip–knee, hip-ankle and knee-ankle cyclograms.

EMG signals were pre-amplified (x10, custom-made system), bandpass filtered (30–1,000 Hz) and amplified (100–5,000×) using a 16-channel amplifier (model 3500; AM Systems, Sequim, WA, United States). As we implanted more than 16 muscles per cat, we obtained data in each locomotor condition twice, one for each connector, as our data acquisition system does not currently allow us to record more than 16 channels simultaneously. EMG data were digitized (2,000 Hz) with a National Instruments card (NI 6032E), acquired with custom-made acquisition software and stored on computer. Although several muscles were implanted, we focused our analysis on the lateral gastrocnemius (LG, ankle extensor/knee flexor; *n* = 7), medial gastrocnemius (MG, ankle extensor/knee flexor; *n* = 7), soleus (SOL, ankle extensor; *n* = 8), vastus lateralis (VL, knee extensor; *n* = 6), biceps femoris anterior (BFA, hip extensor; *n* = 7), iliopsoas (IP, hip flexor; *n* = 6), anterior sartorius (SRT, hip flexor/knee extensor; *n* = 6), semitendinosus (ST, hip extensor/knee flexor; *n* = 7) and biceps femoris posterior (BFP, hip extensor/knee flexor; *n* = 6). We determined burst onsets and offsets by visual inspection from the raw EMG waveforms using a custom-made program. Burst duration was measured from onset to offset. Mean EMG amplitude was measured by integrating the full-wave rectified EMG burst from onset to offset and dividing it by its burst duration. For each cat, we express the mean obtained at a given speed in all three conditions as a percentage of the maximal value obtained in the Intact4 condition. All these analyses were performed on the main burst, although we also qualitatively characterized the presence of a second burst in some muscles.

### Statistical Analysis

We performed all statistical tests with IBM SPSS Statistics 20.0. To determine the effects of locomotor conditions and speed on spatiotemporal and EMG parameters during tied-belt locomotion, we performed a two-factor [(conditions: Intact4, Intact2, Spinal2) × (speeds: from 0.4 to 1.0 m/s)] repeated measures ANOVA. We performed pairwise comparisons if we obtained a significant main effect of condition with no adjustments for multiple comparisons, as discussed in Rothman (1990), Hurteau and Frigon (2018), and Harnie et al. (2019). We did this to avoid type II errors, as discussed in Hurteau and Frigon (2018). If we found a main effect in both factors, we determined if there was a significant interaction. We used a significance level of *P* < 0.05 for statistical significance. We also performed linear regression analyses on burst durations and amplitudes across speeds and measured the slope of the relationship.

## Results

To assess state- and condition-dependent changes in the hindlimb locomotor pattern, we measured and compared kinematic and EMG variables during treadmill locomotion from moderate (0.4 m/s) to high (1.0 m/s) speeds during quadrupedal (Intact4) and hindlimb-only locomotion (Intact2) in intact cats and hindlimb-only locomotion (Spinal2) in spinal cats on a treadmill.

### Spatiotemporal Adjustments With Increasing Speed

**FIGURE 1:**
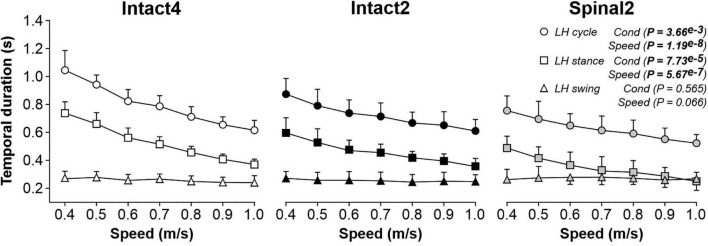
Modulation of temporal parameters during quadrupedal or hindlimb-only locomotion in intact and spinal cats across speeds. Each panel shows cycle, stance and swing durations during Intact4, Intact2 and Spinal2 conditions at seven treadmill speeds. At each speed, we averaged 6–15 cycles per cat. Each data point indicates the mean ± SD for the group (*n* = 9 cats). *P* values comparing conditions (Cond) and speeds are indicated (main effects of repeated-measures ANOVA).

Figure 1 shows cycle and phase durations from 0.4 to 1.0 m/s for the group. We found a significant main effect of condition on cycle and stance durations but not on swing duration. On average, cycle duration was significantly shorter in Spinal2 compared to Intact4 (*P* = 5.75^*e*–3^; 0.17s or 21.7% difference) and Intact2 (*P* = 0.044; 0.09 s or 12.9% difference). Similarly, stance duration was significantly shorter in Spinal2 compared to Intact4 (*P* = 0.021; 0.17s or 32.8% difference) and Intact2 (*P* = 0.019; 0.10s or 23% difference). In accordance with earlier studies (Bélanger et al., 1996; de Leon et al., 1998; Frigon and Rossignol, 2008), we found that cycle and stance durations significantly decreased with increasing speed while swing duration did not change significantly. We also observed a significant interaction for cycle (*P* = 1.68^*e*–8^) and stance (*P* = 1.76^*e*–7^) durations. These results suggest less modulation in both hindlimb–only conditions for cycle duration (% decrease from 0.4 to 1.0 m/s: Intact4 = 41.12%, Intact2 = 30.13%, Spinal2 = 30.83%) and the Intact2 condition for stance duration (% decrease from 0.4 to 1.0 m/s: Intact4 = 49.89%, Intact2 = 40.09%, Spinal2 = 48.79%). In the spinal state, stance duration was equal to or shorter than swing at 0.9 and 1.0 m/s, as shown previously (Frigon et al., 2017; Harnie et al., 2018; Latash et al., 2020). We did not observe this convergence in stance and swing durations in the intact state during quadrupedal and hindlimb-only locomotion.

**FIGURE 2:**
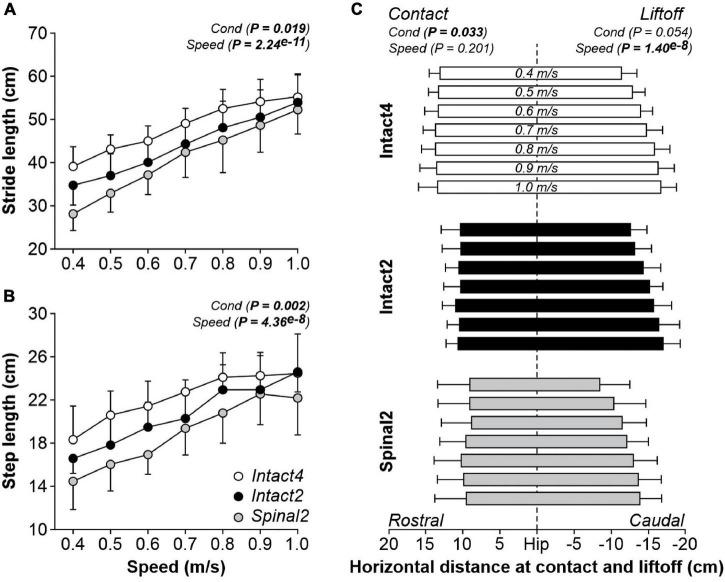
Modulation of spatial parameters during quadrupedal or hindlimb-only locomotion in intact and spinal cats across speeds. Stride length **(A)**, step length **(B)** and the horizontal distance at liftoff/contact **(C)** are shown during Intact4, Intact2 and Spinal2 conditions at seven treadmill speeds. At each speed, we averaged 6–15 cycles per cat. Each data point or bar indicates the mean ± SD for the group (*n* = 9 cats). *P* values comparing conditions (Cond) and speeds are indicated (main effects of repeated-measures ANOVA). Vertical dashed line indicates the zero or hip position.

Stride (Figure 2A) and step (Figure 2B) lengths were significantly different between conditions and significantly increased with speed. Stride length was significantly longer in Intact4 compared to Intact2 (*P* = 0.043, 4.20 cm or 8.7% difference) and Spinal2 (*P* = 0.015, 7.16 cm or 14.8% difference). However, we found no significant differences between Intact2 and Spinal2 (*P* = 0.294). A significant interaction was observed for stride length (*P* = 2.31^*e*–5^), with an apparently greater modulation in the hindlimb–only conditions, particularly in the spinal state (% increase from 0.4 to 1.0 m/s: Intact4 = 41.08%, Intact2 = 55.29%, Spinal2 = 85.26%).

Step length was, on average, significantly longer in Intact4 compared to Spinal2 (*P* = 0.002, 3.32 cm or 14.9% difference). However, we found no significant differences between Intact4 and Intact2 (*P* = 0.080) and between Intact2 and Spinal2 (*P* = 0.059). We found a significant main effect of condition for the horizontal distance at contact but not at liftoff (Figure 2C). On average, the distance at contact was more rostral in the Intact4 condition compared to Intact2 (*P* = 0.009, 2.93 cm or 21.8% difference) and Spinal2 (*P* = 0.048, 3.97 cm or 29.6% difference). We found no significant differences in the horizontal distance at contact between Intact2 and Spinal2 (*P* = 0.519). In intact and spinal cats, the distance of the hindpaw relative to the hip at liftoff was significantly more caudal to the hip with increasing speed while the distance at contact was unaffected by speed (*P* = 0.201).

**FIGURE 3:**
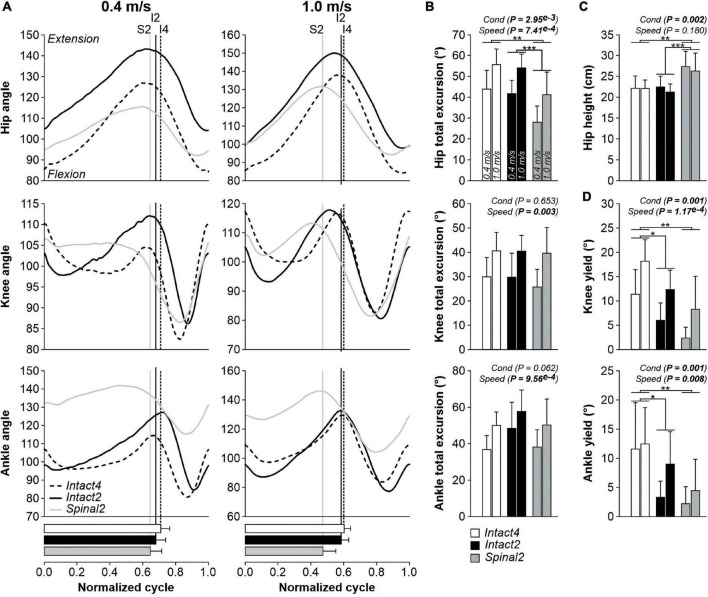
Modulation of joint kinematics during quadrupedal or hindlimb-only locomotion in intact and spinal cats across speeds. **(A)** Angles of the hip, knee and ankle joints as a function of the normalized cycle during Intact4, Intact2 and Spinal2 conditions at 0.4 and 1.0 m/s. (B) Total excursions of the hip, knee and ankle joints at 0.4 and 1.0 m/s. **(C)** Height of the hip marker in the three conditions at 0.4 and 1.0 m/s. **(D)** Yield of the knee and ankle joints at 0.4 and 1.0 m/s. At each speed, 6–15 cycles were averaged per cat. In panel **(A)**, each line represents the mean for the group (*n* = 9 cats). Horizontal bars represent mean stance durations normalized to the cycle and vertical lines indicate paw liftoffs. In panels **(B–D)**, each data bar shows the mean ± SD for the group (*n* = 9 cats). *P* values comparing conditions (Cond) and speeds are indicated (main effects of repeated-measures ANOVA). One, two or three asterisks indicate a significant difference of the pairwise comparison at *P* < 0.05, *P* < 0.01 or *P* < 0.001, respectively.

Figure 3 shows changes in joint angles between conditions at 0.4 m/s and 1.0 m/s for the group (*n* = 9 cats). In all three conditions, the hip, knee, and ankle joints flexed at swing onset followed by extension during mid- to late swing (Figure 3A). At 0.4 m/s, hip flexion began before liftoff in all three conditions. At 1.0 m/s, hip flexion also occurred before liftoff in the intact conditions but coincided with liftoff in the spinal state. At 0.4 m/s, the knee started flexing before swing onset in the Spinal2 condition while in the intact conditions, flexion and swing onsets coincided. The transition to knee extension during swing also occurred relatively earlier during swing in the Intact4 condition compared to the hindlimb-only conditions. At 1.0 m/s, knee flexion onset occurred before swing onset in all three conditions and the transition to extension occurred around mid-stance, although it remained relatively earlier in the Intact4 condition. Across the cycle, the range of angular excursion significantly differed between conditions for hip angle but not for the knee and ankle (Figure 3B). On average, the angular excursion of the hip was smaller in Spinal2 compared to Intact4 (*P* = 0.004, 15.09° or 30.4% difference) and Intact2 (*P* = 0.001, 13.20° or 27.6% difference) conditions. We found no significant differences between the intact conditions (*P* = 0.478). All joint excursions increased significantly with speed (Figure 3B). We also found a significant effect of condition on hip height, with a significantly higher hip height in the Spinal2 condition compared to Intact4 (*P* = 0.007) and Intact2 (*P* = 0.001) (Figure 3C). Speed did not significantly affect hip height. In early stance, the knee and ankle yielded (flexed) before extending. Knee (*P* = 0.001) and ankle (*P* = 0.001) yield significantly differed between conditions and significantly increased with speed (Figure 3D). On average, knee yield was significantly larger in the Intact4 condition compared to Intact2 (*P* = 0.015, 5.60° or 161.1% difference) and Spinal2 (*P* = 0.002, 9.4° or 277% difference). On average, ankle yield was significantly larger in Intact4 compared to Intact2 (*P* = 0.011, 5.9° or 195.3% difference) and Spinal2 (*P* = 0.002, 8.7° or 361.5% difference). When comparing knee and ankle yield in the two hindlimb-only conditions, we found no significant differences.

**FIGURE 4:**
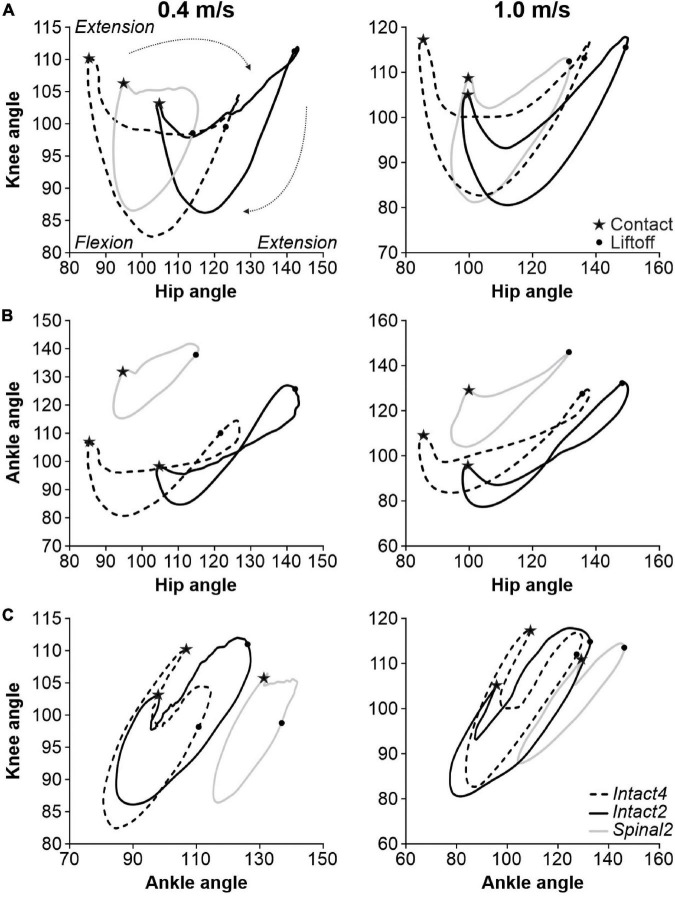
Interjoint coordination during quadrupedal or hindlimb-only locomotion in intact and spinal cats across speeds. **(A)** Knee-hip, **(B)** Ankle-Hip and **(C)** Knee-ankle cyclograms during Intact4, Intact2 and Spinal2 conditions at 0.4 and 1.0 m/s. In panels **(A–C)** and at each speed, 6–15 cycles were averaged per cat. Each line is the mean for the group (*n* = 9 cats). Contact and liftoff are indicated by a star and a dot, respectively. The arrows around the cyclograms indicate the direction of time progression in a clockwise direction.

As described above, transition points from flexion to extension, and vice-versa, could occur at different times in the normalized cycle depending on the condition and the specific joint angle. To assess interjoint coordination, we plotted the different joint angles relative to one another. Figure 4 shows angle-angle plots, or cyclograms, for the three conditions at 0.4 and 1.0 m/s for the group. A positive slope represents simultaneous movement of the two joints in the same direction (e.g., both flexing), while a negative slope represents the two joints moving in opposite direction (e.g., flexion and extension). When the representing curve moves vertically or horizontally, it means that a change in angle is observed in one joint only (Abelew et al., 2000). Please note that the cyclograms have a clockwise direction.

The knee-hip cyclograms displayed a crescent shape at both speeds, with notable differences between states (Figure 4A). In the intact state, the period following contact was characterized by an almost vertical line, with rapid knee flexion and little hip movement. Contact was attenuated by flexion of the knee, as knee extensors contract eccentrically. In the spinal state, this knee flexion was absent. During weight bearing in the Intact4 condition, the hip extends without knee movement, as shown by the horizontal line. The propulsion phase at the end of stance phase is characterized by concomitant hip and knee extension (positive slope). Interestingly, in the Intact2 condition after the knee yield, stance is characterized by extension of the hip and knee until liftoff. In the spinal state, the knee angle changes less during stance, particularly at 0.4 m/s. After liftoff, the hip and knee both flex in all three conditions. After reaching maximum flexion, the knee extends in preparation for the next foot placement. During this period, the hip smoothly continues to flex in the intact state while it extends slightly in the spinal state due to an overshoot in forward hip movement at the end of swing (i.e., the hip must extend to place the paw on the treadmill).

The ankle-hip movement was characterized by a figure eight-like trajectory in the intact state (Figure 4B). The period after contact consists of ankle flexion (ankle yield) with a slight hip extension, particularly in Intact4. Ankle yield is mainly absent in Spinal2. At the end of stance, the ankle extends rapidly in the intact state while the hip remains stationary followed after liftoff by hip and ankle flexion, creating the figure eight shape. In the spinal state, rapid ankle extension did not occur at the end of stance and the ankle-hip movement maintained a crescent shape. Hip flexion reaches its maximal value before contact in the spinal state and the hip must extend before placement. In the intact state, in late swing, the ankle extends while the hip continues to flex before contact. Concomitant knee and ankle yield after contact is pronounced in Intact4 compared to hindlimb-only locomotion, particularly when compared to Spinal2 (Figure 4C).

### Electromyography Adjustments With Increasing Speed

**FIGURE 5:**
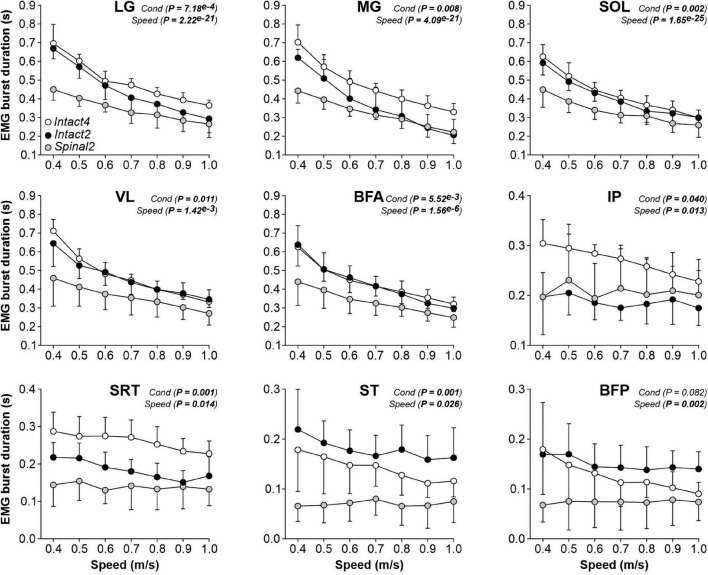
Modulation of EMG burst durations during quadrupedal or hindlimb-only locomotion in intact and spinal cats across speed. The figure shows burst durations in nine hindlimb muscles during Intact4, Intact2 and Spinal2 conditions at seven treadmill speeds. At each speed, 6–15 cycles were averaged per cat. Each data point indicates the mean ± SD for the group. *P* values comparing conditions (Cond) and speeds are indicated (main effects of repeated-measures ANOVA). LG, lateral gastrocnemius (*n* = 7 cats); MG, medial gastrocnemius (*n* = 7 cats); SOL, soleus (*n* = 8 cats); VL, vastus lateralis (*n* = 6 cats); BFA, biceps femoris anterior (*n* = 7 cats); IP, iliopsoas (*n* = 6 cats); SRT, anterior sartorius (*n* = 6 cats); ST, semitendinosus (*n* = 7 cats); BFP, biceps femoris posterior (*n* = 6 cats).

To determine if the locomotor conditions and/or state affected muscle activity, we measured EMG activity (duration, amplitude and phasing) in the three conditions. We found a significant effect of condition on EMG burst durations for all extensor muscles (LG, MG, SOL, VL and BFA; Figure 5, first five panels). Table 1 summarizes significant differences in EMG burst durations between conditions. Only the MG burst duration was significantly longer in Intact4 compared to Intact2, while all extensor muscles (LG, MG, SOL, VL and BFA) were significantly longer in Intact4 compared to Spinal2. When comparing the two hindlimb-only conditions, we found that extensor burst durations in the intact state were significantly longer for 3 muscles (LG, SOL and BFA) compared to the spinal state. We also found a significant interaction for all extensor muscles (P_*LG*_ = 3.54^*e*–5^, P_*MG*_ = 3.21^*e*–5^, P_*SOL*_ = 1.12^*e*–5^, P_*VL*_ = 5.57^*e*–9^, P_*BFA*_ = 1.45^*e*–3^), with less modulation in the spinal state from 0.4 to 1.0 m/s.

**TABLE 1:** Effect of locomotor condition and state on EMG burst durations.

		*Intact2*	*Spinal2*
LG	*Intact4*	*P* = 0.103	*P* = 5.53^*e*–4^*
	*Intact2*		*P* = 0.007*
MG	*Intact4*	*P* = 0.036*	*P* = 2.12^*e*–6^*
	*Intact2*		*P* = 0.158
SOL	*Intact4*	*P* = 0.400	*P* = 0.007*
	*Intact2*		*P* = 0.005*
VL	*Intact4*	*P* = 0.399	*P* = 0.017*
	*Intact2*		*P* = 0.067
BFA	*Intact4*	*P* = 0.843	*P* = 0.008*
	*Intact2*		*P* = 0.001*
IP	*Intact4*	*P* = 0.006*	*P* = 0.108
	*Intact2*		*P* = 0.057
SRT	*Intact4*	*P* = 0.002*	*P* = 0.004*
	*Intact2*		*P* = 0.174
ST	*Intact4*	*P* = 0.078	*P* = 0.012*
	*Intact2*		*P* = 0.007*

*The table shows P values comparing EMG burst durations during quadrupedal (Intact4) and hindlimb-only (Intact2) locomotion in the intact state and hindlimb-only (Spinal2) locomotion in the spinal state (n = 9 cats). For each speed and condition, we averaged 6–15 cycles per cat.*

*LG, lateral gastrocnemius; MG, medial gastrocnemius; SOL, soleus; VL, vastus lateralis; BFA, biceps femoris anterior; IP, iliopsoas; SRT, anterior sartorius; ST, semitendinosus.*

*An asterisk indicates a significant difference of the pairwise comparison at P < 0.05.*

We found a significant effect of locomotor conditions on EMG burst durations for three muscles with a main activation during the swing phase (IP, SRT and ST) but not for BFP (Figure 5 last four panels). It should be noted that SRT and ST are not pure flexors. On average, IP and SRT burst durations were significantly longer in the Intact4 condition compared to the Intact2 condition (Table 1). SRT and ST burst durations were significantly longer in Intact4 compared to Spinal2. Only the ST burst duration was significantly longer in the Intact2 condition compared to Spinal2. We found a significant effect of speed on burst durations for IP, SRT, ST and BFP muscles. We also observed a significant interaction for IP (*P* = 0.005), SRT (*P* = 0.011) and ST (*P* = 0.006), with less modulation in the spinal state from 0.4 to 1.0 m/s.

**FIGURE 6:**
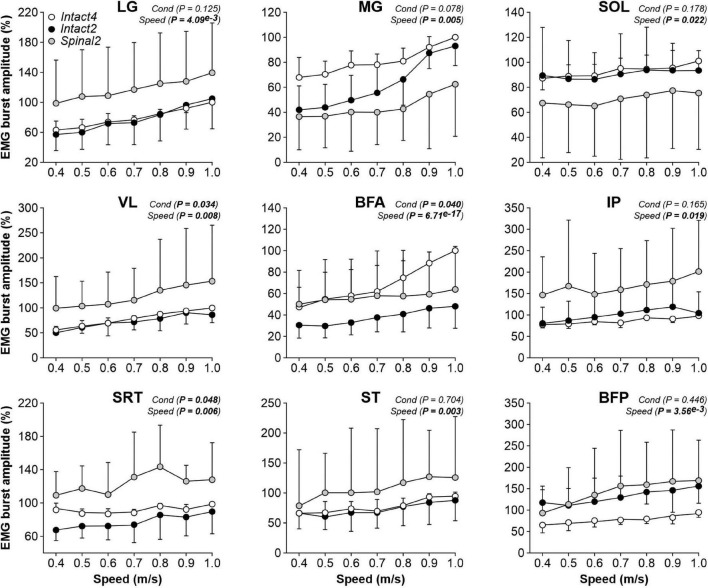
Modulation of EMG burst amplitudes during quadrupedal or hindlimb-only locomotion in intact and spinal cats across speeds. The figure shows burst amplitudes from nine hindlimb muscles during Intact4, Intact2 and Spinal2 conditions at seven treadmill speeds. At each speed, 6–15 cycles were averaged per cat. For each cat, the mean obtained at a given speed in all three conditions was expressed as a percentage of the maximal value obtained in Intact4. Each data point indicates the mean ± SD for the group. *P* values comparing conditions (Cond) and speeds are indicated (main effects of repeated-measures ANOVA). For muscle abbreviations, see Figure 5 legend.

We found a significant effect of condition on EMG burst amplitude for VL and BFA muscles but not for the three triceps surae muscles (LG, MG and SOL; Figure 6, first five panels). However, when comparing specific conditions, only BFA showed a significant difference (*P* = 0.011), with a larger BFA EMG amplitude in Intact4 compared to Intact2. We also observed a significant interaction for BFA (*P* = 1.07^*e*–6^), with less modulation in the hindlimb–only conditions, particularly in the spinal state (% increase from 0.4 to 1.0 m/s, Intact4 = 110.23%, Intact2 = 57.72%, Spinal2 = 27.44%). For flexors, we found a significant effect of condition on EMG burst amplitude for SRT only and not for IP, ST and BFP (Figure 6, last four panels). This is likely due to the high variance between conditions. For instance, at 0.4 m/s in the LG muscle, 3 cats showed a twofold greater EMG burst amplitude in the spinal state compared to the intact state, whereas the opposite was observed for the 4 other cats analyzed.

To determine the modulation of burst durations and amplitudes with increasing speed, we performed linear regressions and measured the slope. We found a significant effect of speed on burst durations for all muscles except SRT (Table 2, left part). We found that the slope values of the burst duration/speed relationship were significantly greater in Intact4 compared to Spinal2 in all muscles that showed a main effect of speed and only greater than Intact2 for IP. Slope values of the burst duration/speed relationship in Intact2 were significantly greater than Spinal2 for all extensors (LG, MG, SOL, VL and BFA) but not the muscles with a main flexor activation (ST, BFP). We found a significant effect of speed on burst amplitudes for 4 muscles (MG, VL, BFA and ST); Table 2, right part). In these 4 muscles, slope values were significantly greater in Intact4 compared to Intact2 for VL and BFA and smaller in MG. The slope was also smaller in Intact4 when compared to Spinal2, but only for ST. When comparing the two hindlimb-only conditions, slope values were significantly greater in Intact2 compared to Spinal2 for MG but smaller for VL and ST. Thus, the spinal state weakens the linear modulation of burst durations with increasing speed, particularly in extensors, while the modulation of burst amplitudes is affected by both condition and state and is muscle specific.

**TABLE 2:** Effect of speed on EMG burst durations and amplitudes in the three conditions.

Burst duration					Burst amplitude				
		*Slope value*	*Intact2*	*Spinal2*			*Slope value*	*Intact2*	*Spinal2*
LG (*P* = 0.003)	*Intact4*	−0.052 ± 0.013	*P* = 0.268	*P* = 0.029*	LG (*P* = 0.51)	*Intact4*	6.176 ± 2.304		
	*Intact2*	−0.061 ± 0.015		*P* = 0.002*		*Intact2*	8.144 ± 3.843		
	*Spinal2*	−0.030 ± 0.014				*Spinal2*	6.389 ± 4.682		
MG (*P* = 0.005)	*Intact4*	−0.058 ± 0.015	*P* = 0.268	*P* = 0.022*	MG (*P* = 0.017)	*Intact4*	5.108 ± 2.027	*P* = 0.013*	*P* = 0.497
	*Intact2*	−0.066 ± 0.012		*P* = 0.017*		*Intact2*	9.183 ± 2.619		*P* = 0.050*
	*Spinal2*	−0.036 ± 0.016				*Spinal2*	4.130 ± 4.067		
SOL (*P* = 0.002)	*Intact4*	−0.051 ± 0.007	*P* = 0.365	*P* = 0.010*	SOL (*P* = 0.69)	*Intact4*	2.150 ± 1.536		
	*Intact2*	−0.047 ± 0.011		*P* = 0.016*		*Intact2*	1.188 ± 2.932		
	*Spinal2*	−0.030 ± 0.012				*Spinal2*	1.969 ± 1.942		
VL (*P* = 0.0006)	*Intact4*	−0.057 ± 0.013	*P* = 0.073	*P* = 0.001*	VL (*P* = 0.50)	*Intact4*	7.515 ± 2.110	*P* = 0.013*	*P* = 0.497
	*Intact2*	−0.046 ± 0.013		*P* = 0.024*		*Intact2*	5.812 ± 1.965		*P* = 0.050*
	*Spinal2*	−0.029 ± 0.014				*Spinal2*	9.775 ± 12.230		
BFA (*P* = 0.050)	*Intact4*	−0.053 ± 0.014	*P* = 0.481	*P* = 0.038*	BFA (*P* = 0.003)	*Intact4*	8.605 ± 3.538	*P* = 0.021*	*P* = 0.016*
	*Intact2*	−0.046 ± 0.013		*P* = 0.047*		*Intact2*	3.352 ± 2.726		*P* = 0.240
	*Spinal2*	−0.031 ± 0.014				*Spinal2*	1.948 ± 1.913		
IP (*P* = 0.012)	*Intact4*	−0.013 ± 0.008	*P* = 0.035*	*P* = 0.025*	IP (*P* = 0.32)	*Intact4*	3.373 ± 1.367		
	*Intact2*	−0.003 ± 0.005		*P* = 0.207		*Intact2*	5.455 ± 3.699		
	*Spinal2*	−0.003 ± 0.007				*Spinal2*	7.529 ± 7.874		
SRT (*P* = 0.108)	*Intact4*	−0.010 ± 0.007			SRT (*P* = 0.33)	*Intact4*	1.413 ± 0.975		
	*Intact2*	−0.011 ± 0.008				*Intact2*	3.618 ± 3.742		
	*Spinal2*	−0.002 ± 0.005				*Spinal2*	3.811 ± 4.008		
ST (*P* = 0.050)	*Intact4*	−0.011 ± 0.011	*P* = 0.584	*P* = 0.023*	ST (*P* = 0.018)	*Intact4*	5.132 ± 2.134	*P* = 0.544	*P* = 0.038*
	*Intact2*	−0.008 ± 0.010		*P* = 0.095		*Intact2*	4.027 ± 2.667		*P* = 0.023*
	*Spinal2*	−0.001 ± 0.004				*Spinal2*	9.619 ± 3.613		
BFP (*P* = 0.042)	*Intact4*	−0.013 ± 0.012	*P* = 0.230	*P* = 0.041*	BFP (*P* = 0.11)	*Intact4*	4.139 ± 3.023		
	*Intact2*	−0.005 ± 0.008		*P* = 0.074		*Intact2*	7.420 ± 3.875		
	*Spinal2*	−0.001 ± 0.002				*Spinal2*	12.818 ± 9.303		

*The table shows mean slope values ± standard deviations obtained by performing a linear regression analysis between burst durations or amplitudes as a function of speed. In the brackets next to the muscle is the P value of the main effect of condition (one-factor ANOVA). If a significant main effect was found, we performed pairwise comparisons and the P values are indicated comparing the three conditions.*

*LG, lateral gastrocnemius; MG, medial gastrocnemius; SOL, soleus; VL, vastus lateralis; BFA, biceps femoris anterior; IP, iliopsoas; SRT, anterior sartorius; ST, semitendinosus; BFP, biceps femoris posterior.*

*An asterisk indicates a significant difference of the pairwise comparison at P < 0.05.*

**FIGURE 7:**
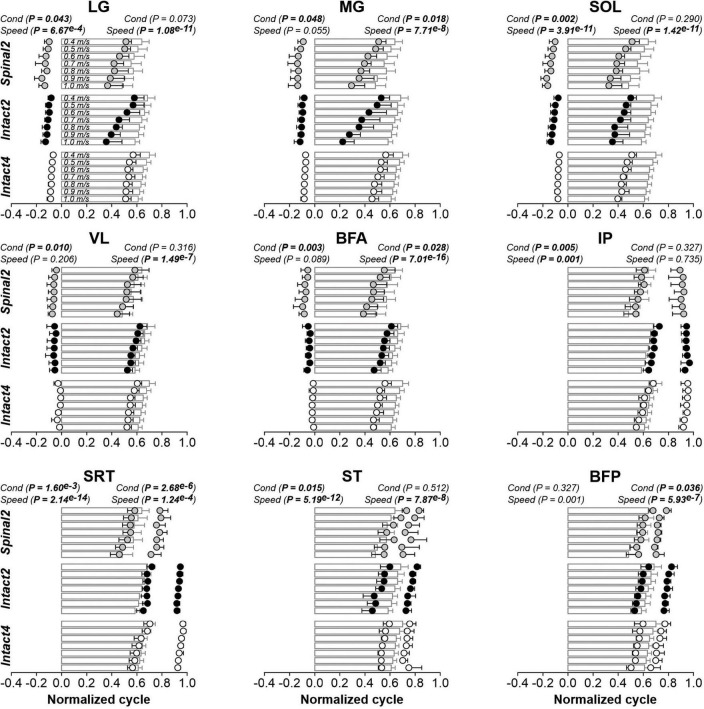
Modulation of EMG phasing during quadrupedal or hindlimb-only locomotion in intact and spinal cats across speeds. The figure shows burst onsets and offsets from nine hindlimb muscles normalized to the cycle during Intact4, Intact2 and Spinal2 conditions at seven treadmill speeds. At each speed, 6–15 cycles were averaged per cat. Data points represent burst onsets and offsets. Horizontal bars represent averaged stance durations normalized to stance onset. Each data point or bar indicates the mean ± SD for the group. *P* values comparing conditions (Cond) and speeds are indicated (main effects of repeated-measures ANOVA). For muscle abbreviations, see Figure 5 legend.

Locomotor condition significantly affected the phasing of EMG burst onsets for all extensor (LG, MG, SOL, VL and BFA) and flexor (IP, SRT and ST) muscles, except for BFP (Figure 7 and Table 3). For extensors, burst onsets occurred, on average, significantly later in Intact4 compared to Spinal2 for all muscles, except for MG. We found no significant differences between the two hindlimb-only conditions. Interestingly, although there was a significant difference between conditions for IP and SRT, the onsets remained in phase with liftoff. On the other hand, while the onset of ST and BFP occurred earlier than liftoff in the intact state, it occurred after liftoff in the spinal state. As speed increased, burst onsets occurred significantly earlier at faster speeds for LG, SOL, IP, SRT, ST and BFP but not for MG, VL and BFA.

**TABLE 3:** Effect of locomotor conditions and state on EMG phasing.

		*Intact2*_*Onset*_	*Spinal2*_*Onset*_	*Intact2*_*Offset*_	*Spinal2*_*Offset*_
LG	*Intact4*	*P* = 0.043*	*P* = 0.035*		
	*Intact2*		*P* = 0.365		
MG	*Intact4*	*P* = 0.074	*P* = 0.059	*P* = 0.017*	*P* = 0.004*
	*Intact2*		*P* = 0.245		*P* = 0.697
SOL	*Intact4*	*P* = 0.017*	*P* = 0.005*		
	*Intact2*		*P* = 0.125		
VL	*Intact4*	*P* = 0.075	*P* = 0.009*		
	*Intact2*		*P* = 0.444		
BFA	*Intact4*	*P* = 0.006*	*P* = 0.014*	*P* = 0.034*	*P* = 0.306
	*Intact2*		*P* = 0.081		*P* = 0.019*
IP	*Intact4*	*P* = 0.006*	*P* = 0.257		
	*Intact2*		*P* = 0.025*		
SRT	*Intact4*	*P* = 0.021*	*P* = 0.014*	*P* = 0.136	*P* = 3.55^*e*–4^*
	*Intact2*		*P* = 0.001*		*P* = 0.001*
ST	*Intact4*	*P* = 0.001*	*P* = 0.031*		
	*Intact2*		*P* = 0.046*		
BFP	*Intact4*			*P* = 0.002*	*P* = 0.881
	*Intact2*				*P* = 0.013*

*The table shows P values comparing EMG burst phasing during quadrupedal (Intact4) and hindlimb-only (Intact2) locomotion in the intact state and hindlimb-only (Spinal2) locomotion in the spinal state (n = 9 cats). For each speed and condition, we averaged 6–15 cycles per cat. An empty box indicates no significant main effect of the ANOVA. LG, lateral gastrocnemius; MG, medial gastrocnemius; SOL, soleus; VL, vastus lateralis; BFA, biceps femoris anterior; IP, iliopsoas; SRT, anterior sartorius; ST, semitendinosus; BFP, biceps femoris posterior.*

*An asterisk indicates a significant difference of the pairwise comparison at P < 0.05.*

We found a significant effect of conditions on EMG burst offsets for two (MG and BFA) of five extensor muscles and two (SRT and BFP) of four flexor muscles. Table 3 shows *P* values for the pairwise comparisons between conditions. The MG offset occurred later in Intact4 compared to the two hindlimb-only conditions, with no significant differences between hindlimb-only conditions. The SRT offset did not significantly differ between intact conditions, but occurred earlier in the spinal condition compared to the two intact conditions (Intact4, *P* = 3.55^*e*–3^ or 24.47% difference; Intact2, *P* = 0.019 or 22.42% difference). Offsets occurred significantly earlier at faster speeds for LG (*P* = 1.08^*e*–11^), MG (*P* = 7.71^*e*–8^), SOL (*P* = 1.42^*e*–11^), VL (*P* = 1.49^*e*–7^), BFA (*P* = 7.01^*e*–16^), SRT (*P* = 1.24^*e*–4^), ST (*P* = 7.87^*e*–8^) and BFP (*P* = 5.93^*e*–7^), but not IP (*P* = 0.735).

### The Appearance of Double Bursts in Some Muscles

**FIGURE 8:**
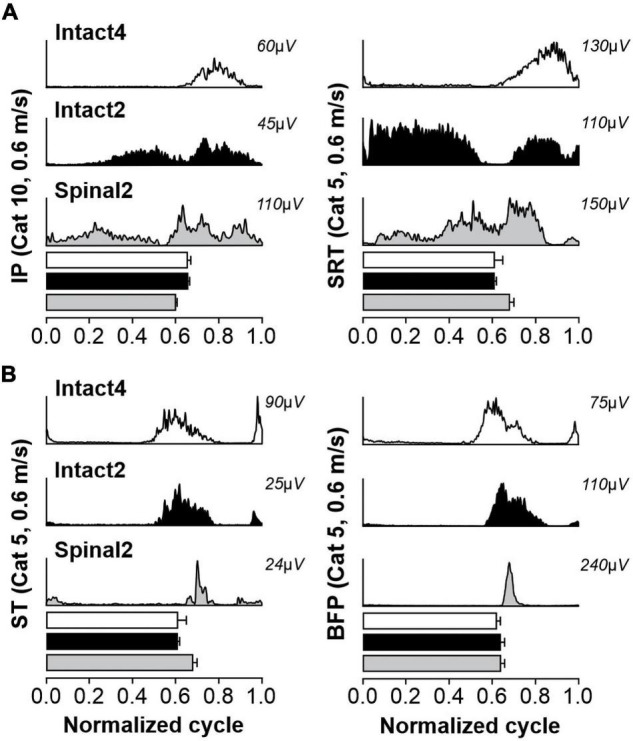
Second bursts of activity in muscles with a flexor action at the hip and knee joints during quadrupedal or hindlimb-only locomotion in intact and spinal cats. The figure shows averaged rectified muscle activity normalized to the cycle for selected muscles during Intact4, Intact2 and Spinal2 conditions at 0.6 m/s for Cats 5 and 10: in **(A)** IP and SRT and **(B)** ST and BFP muscles. Horizontal bars represent averaged stance durations normalized to the cycle. For each muscle, 10–15 cycles were averaged. IP, iliopsoas; SRT, anterior sartorius; ST, semitendinosus; BFP, biceps femoris posterior.

It is well known that some muscles can display two bursts during the step cycle and that these can be influenced by speed and the type of locomotion (Halbertsma, 1983; Smith et al., 1993; de Leon et al., 1998; Desrochers et al., 2019). One study reported the appearance of a second burst in IP activity at a speed of 0.4 m/s in the stance phase of the Intact2 (incidence = 92%) and Spinal2 (incidence = 85%) conditions in cats (de Leon et al., 1998). During normal level quadrupedal locomotion, only a single burst of activity generally occurs in the IP, corresponding approximately to the swing phase. Here, we wanted to determine if a second burst appeared in other muscles with flexor actions in hip and knee joints and if this was affected by speed. For instance, the SRT is a synergist of the IP for hip flexion. In the Intact4 condition, we observed that the incidence of the second burst during stance was low in the IP (incidence = 6%) and SRT (incidence = 3%) muscles at 0.4 m/s (Figure 8A) and not influenced by speed (Table 4). In contrast, we consistently observed a period of activity during stance in the hindlimb-only conditions for IP and SRT (Table 4). The incidence was lower in the Spinal2 condition compared to Intact2 and it appeared to be influenced in the former by increasing speed, albeit not consistently.

**TABLE 4:** Effect of locomotor conditions and state on appearance of double bursts in flexor muscles.

		0.4	0.5	0.6	0.7	0.8	0.9	1.0
IP	*Intact4*	6% (4/67)	0% (0/72)	0% (0/82)	0% (0/78)	0% (0/79)	0% (0/77)	0% (0/73)
	*Intact2*	97% (69/71)	96% (82/85)	90% (76/84)	95% (75/79)	97% (86/89)	94% (68/72)	90% (64/71)
	*Spinal2*	47% (37/79)	49% (34/70)	54% (44/81)	54% (41/76)	49% (34/69)	60% (40/67)	69% (45/65)
SRT	*Intact4*	3% (2/67)	0% (0/80)	0% (0/85)	0% (0/88)	0% (0/86)	0% (0/86)	0% (0/85)
	*Intact2*	100% (74/74)	100% (84/84)	98% (80/82)	100% (80/80)	98% (83/85)	100% (76/76)	94% (73/78)
	*Spinal2*	30% (23/76)	73% (51/70)	35% (25/72)	49% (35/71)	63% (42/67)	64% (37/58)	53% (30/57)
ST	*Intact4*	65% (56/86)	73% (58/79)	91% (89/98)	90% (76/84)	92% (81/88)	100% (90/90)	100% (67/67)
	*Intact2*	91% (75/82)	83% (68/82)	98% (90/92)	100% (91/91)	94% (81/86)	99% (74/75)	100% (96/96)
	*Spinal2*	28% (23/83)	39% (37/94)	52% (41/79)	58% (44/76)	46% (36/78)	67% (51/76)	69% (59/86)
BFP	*Intact4*	54% (37/69)	68% (50/74)	88% (74/84)	82% (65/79)	91% (73/80)	92% (78/85)	93% (74/80)
	*Intact2*	96% (69/72)	97% (83/86)	100% (85/85)	98% (78/80)	100% (90/90)	99% (72/73)	97% (70/72)
	*Spinal2*	7% (6/83)	9% (6/70)	4% (3/80)	0% (0/76)	4% (3/69)	4% (3/71)	3% (2/70)

*The table shows the incidence of a second burst in flexor muscles during Intact4, Intact2, and Spinal2 conditions at seven treadmill speeds (n = 9 cats). For each muscle, the number of second bursts during the trial is indicated as a percentage of the total number of cycles within the trial.*

*IP, iliopsoas; SRT, anterior sartorius; ST, semitendinosus; BFP, biceps femoris posterior.*

Several studies have observed the presence of a second burst in the ST muscle and its close synergist BFP (hip extensors/knee flexors) just before paw contact during quadrupedal locomotion (Chanaud et al., 1991; Smith et al., 1993; Desrochers et al., 2019; Klishko et al., 2021). Although not always present at slow speeds (Smith et al., 1993), this burst decelerates forward movement of the limb before paw contact, particularly at faster speeds (Wisleder et al., 1990; Smith et al., 1993; Pratt et al., 1996). This second ST burst has also been shown in spinal cats (Desrochers et al., 2019). In the Intact4 condition, the second burst in ST and BFP was present at the slowest tested speed of 0.4 m/s with an incidence of 65 and 54%, respectively. At the fastest speed tested, the incidence increased to 100 and 93%, respectively (Figure 8B and Table 4). In the Intact2 condition, the incidence of the second burst in ST and BFP started high, with 91 and 96% at 0.4 m/s and was maintained with increasing speed. In the Spinal2 condition, the incidence of the second burst in ST increased with speed, whereas the second burst in BFP was mostly absent across speeds.

## Discussion

Although a few studies have compared hindlimb-only locomotion in intact and spinal cats during forward locomotion (de Guzman et al., 1991; de Leon et al., 1998), as discussed below, they mainly focused on a few variables at a single speed and/or they did not use the same animal as its own control. Here, we extend those findings, by comparing several additional spatiotemporal variables along with joint kinematics and EMG activity of several muscles across a range of speeds before and after spinal transection in the same animal. We also report novel findings on interjoint coordination in spinal cats not previously explored.

### State- and Condition-Dependent Changes in Spatiotemporal and Kinematic Variables

When evaluating hindlimb-only forward locomotion in spinal cats, studies have mostly compared the hindlimb pattern with the one obtained during quadrupedal locomotion in intact cats (Forssberg et al., 1980b; Smith et al., 1982; Bélanger et al., 1996; Rossignol et al., 2001; Frigon and Rossignol, 2008; Barrière et al., 2010; Kuczynski et al., 2017; Desrochers et al., 2019). Here, we show that spatiotemporal adjustments of the hindlimb pattern in chronic spinal adult cats resemble those found in intact animals, especially when compared with the hindlimb-only intact condition. For example, cycle and stance durations in Spinal2 were closer to values observed in Intact2 compared to Intact4. Stride and step lengths were shorter in the spinal state compared to Intact4 but there were no significant differences between the two hindlimb-only conditions. Another important parameter during locomotion is the position of the paw at contact (Halbertsma, 1983; Klishko et al., 2014). Studies have reported a less rostral position of the paw at contact relative to the hip in spinal cats (Bélanger et al., 1996; Frigon and Rossignol, 2008). Here, we found a significant difference when comparing Spinal2 with Intact4 but not when comparing the two hindlimb-only conditions, indicating that the hindlimb-only condition itself influences paw placement at contact. Overall, the results of the present study indicate that some spatiotemporal adjustments of the hindlimb pattern can be attributed to the spinal transection (change in state) while other changes are because the forelimbs are stationary.

Note that in the hindlimb-only conditions, the forelimbs are slightly raised on a platform and this potentially increases the load on the hindlimbs. During normal quadrupedal locomotion, cats bear a greater percentage of their bodyweight on their forelimbs because of the weight of the head and neck (Frigon et al., 2021), while in the hindlimb-only conditions with elevated forelimbs, there is a caudal shift of the body’s center of mass and consequently more weight on the hindlimbs. However, our results do not show an increase in the duration of stance consistent with increased load on the hindlimb (Duysens and Pearson, 1980; Conway et al., 1987; Bouyer and Rossignol, 2003; Frigon, 2017). Moreover, hindlimb extensor amplitude was not different between the intact and spinal states. One study in spinal rats showed an increase in cycle and extensor muscle duration when the animal was moved from a horizontal to a vertical position, which facilitated the expression of spinal hindlimb-only locomotion (Sławińska et al., 2012). In our study, we attempted to place the animals in a similar position in the three conditions, to avoid shifting weight to the hindlimbs in the hindlimb-only conditions. Another biomechanical factor to consider when comparing normal quadrupedal and hindlimb-only locomotion at the same speed is that in quadrupedal locomotion, the forelimbs decelerate the forward movement of the body on average in a cycle of steady-state locomotion – their braking impulse of the horizontal ground reaction force is greater than the propulsive impulse (Farrell et al., 2014). Therefore, to maintain a constant speed, the hindlimbs must accelerate the body in each cycle, and their propulsive impulse is greater than the braking impulse during quadrupedal locomotion (Farrell et al., 2014). During constant-speed treadmill locomotion of bipeds, the braking and propulsive impulses are the same (Nilsson and Thorstensson, 1989; Stover et al., 2018). Thus, during Intact2 locomotion, the hindlimbs should have smaller forward propulsion demands and more braking demands compared to the intact quadrupedal condition.

As in other studies (Smith et al., 1982; Bélanger et al., 1996), we found an absence of ankle and knee yield in early stance in spinal cats. Interestingly, ankle and knee yields did not significantly differ between the two hindlimb-only conditions and were significantly smaller compared to the Intact4 condition. Smith et al. (1982) argued that the lack of yield could result from an earlier pre-contact activation of extensor muscles. Consistent with this, the onset of extensor muscles appeared earlier in the Intact2 and Spinal2 conditions (Figure 7). This earlier activation of extensors might reduce or prevent knee and ankle yield after paw contact (Smith et al., 1982).

Studies in spinal kittens (Forssberg, 1979; Forssberg et al., 1980a) and adult spinal cats (Barbeau and Rossignol, 1987; Bélanger et al., 1996) reported that the coupling between the hip-knee and knee-ankle joints were similar to those observed during quadrupedal locomotion in the intact state. We confirm those findings, showing similar cyclogram shapes across conditions and states. However, we found a different coupling between the hip and ankle angles in spinal cats, with an absence of the characteristic figure-eight shape, compared to the two conditions in the intact state (Figure 4C). In the intact state, the ankle joint rapidly extended at the end of stance while the hip angle varied little. In the spinal state, the transition from stance to swing occurred without this rapid extension of the ankle, which can reduce the force of propulsion.

### State- and Condition-Dependent Changes in Hindlimb Muscle Activity

It is well established that EMG activity obtained during quadrupedal locomotion in intact cats and hindlimb-only locomotion in spinal cats is qualitatively similar, despite some differences in duration, amplitude and phasing (Bélanger et al., 1996; Frigon and Rossignol, 2008). Here, we found that muscle activity patterns across speeds after spinal transection were similar to those of the intact state, particularly when compared to the Intact2 condition. For example, as reported by de Guzman et al. (1991), we observed a shorter burst duration in the MG muscle in the Intact2 and Spinal2 conditions compared to Intact4. We also observed this for the LG muscle but not for the other extensor muscles analyzed (SOL, VL, BFA). As mentioned, this cannot be explained by more load on the hindlimbs during hindlimb-only locomotion, which would have increased extensor burst durst duration. A decrease in extensor amplitude and an increase in flexor amplitude has been reported during hindlimb-only locomotion in the spinal state compared to quadrupedal locomotion (Bélanger et al., 1996; de Leon et al., 1998; Frigon and Rossignol, 2008) or hindlimb-only locomotion in the intact state (de Leon et al., 1998). These results could be explained by differences in mechanical demands on the hindlimbs between the hindlimb-only and quadrupedal conditions, as discussed above. In the present study, we found no significant differences, possibly because of the high inter-animal variability (see also (Frigon and Rossignol, 2008), highlighted by large standard deviations.

Studies have reported activation of the triceps surae muscles (LG, MG, SOL) before paw contact during quadrupedal locomotion in intact cats (Engberg and Lundberg, 1969; Gregor et al., 2006; Rossignol, 2011; Desrochers et al., 2019), while VL and BFA muscles activate at or around contact. After spinalization, these five extensor muscles become active earlier (Frigon and Rossignol, 2008; Desrochers et al., 2019). We also found an earlier onset of the five extensor muscles in the Intact2 condition, similar to the spinal state, when compared to Intact4. Some studies have attributed this pre-contact EMG to a centrally-generated mechanism (i.e., by the spinal locomotor CPG) (Engberg and Lundberg, 1969; Rossignol, 2011) while other studies have demonstrated a role of length feedback from hip extensors in late stance (McVea et al., 2005; Akay et al., 2014; Markin et al., 2016). As stated, the hindlimbs in late stance overshoot in spinal cats, with peak hip flexion occurring before contact. This greater hip flexion before contact likely activates length feedback from hip extensors contributing to an earlier activation of extensor muscles.

At the stance-to-swing transition in the intact state, the knee flexor/hip extensor muscles ST and BFP activate before the hip flexor muscles IP and SRT, ensuring that the knee flexes and the hip slightly extends before the hip flexes (Figure 4A). This allows the foot to lift from the ground before the limb moves forward. After spinalization, hip flexors activate before knee flexors, which can lead to paw drag (Martinez et al., 2011). In our study, we did not observe paw drag because the majority of cats stepped with their knees extended. The change in hip and knee flexor timing is state-related, as proper timing was observed in both intact conditions.

During level quadrupedal treadmill locomotion, IP and SRT muscles display a single period of activity during the swing phase (Figure 8A; Rasmussen et al., 1978; Halbertsma, 1983; de Leon et al., 1998; Desrochers et al., 2019). However, during downslope walking, the IP and SRT become active during stance (Smith et al., 1998; Klishko et al., 2021) to better control the descent (Gregor et al., 2006). The SRT and IP muscles also display a second burst of activity during the stance phase in the spinal state (Bélanger et al., 1996; de Leon et al., 1998; Rossignol and Bouyer, 2004) and in the Intact2 condition (Figure 8A; de Leon et al., 1998). Thus, the second period of activity in hip flexors appears related to the hindlimb-only condition and not the spinal state and can be related to an increased demand of the hindlimbs for braking compared to the quadrupedal condition. The ST, a two-joint muscle, displays a typical two-burst pattern during quadrupedal locomotion (Smith et al., 1993; Markin et al., 2012) and hindlimb-only locomotion in spinal cats (Figure 8B; Bélanger et al., 1996; Rossignol and Bouyer, 2004; Frigon and Rossignol, 2008). Here, we show, as in de Leon et al. (1998), that the incidence of the second bursts is almost maximal in the Intact2 condition. The BFP muscle, a close synergist of the ST muscle, also displayed two periods of activity in the intact and spinal states. However, the incidence of a second burst was considerably higher in both intact conditions (>50%) compared to the spinal state (<10%). This indicates that state, and not locomotor condition, affects burst patterns in the BFP muscle. It also highlights a differential control of activity in the ST and BFP muscles, possibly in their actions for thigh adduction-abduction and supination-pronation, as they are close synergists for knee flexion-hip extension.

### Control of Locomotor Adjustments With Speed

During normal locomotion in cats and humans, an increase in speed is accompanied by a significant reduction of stance phase duration while swing phase duration remains relatively the same (Goslow et al., 1973; Wetzel and Stuart, 1976; Halbertsma, 1983; Nilsson et al., 1985; Gossard et al., 2011; Frigon et al., 2013, 2014). These cycle/phase duration relationships were maintained across locomotor conditions in the intact and spinal state. However, we observed less modulation in cycle and stance duration in the hindlimb-only conditions. Another notable difference in spinal cats is the convergence of swing durations around 0.9 m/s that was not observed in the intact state during quadrupedal and hindlimb-only locomotion. Latash et al. (2020) recently investigated this convergence by mathematically modelling the organization of the spinal locomotor CPG. They combined the half-center concept and the flexor-driven concept and suggested that CPG operation is state-dependent and particularly speed-dependent. With increasing speed, CPG operation switches from a flexor-driven rhythmicity to classical half-center oscillations with a balanced flexor-extensor pattern. In spinal cats, this switch is made at lower speeds than in the intact state.

In all three conditions and both states, spatial parameters changed similarly with increasing speed. Step and stride lengths increased, the relative distance of the hindpaw at contact remained invariant and the distance at liftoff became progressively more caudal. The angular excursions of the hip, knee and ankle increased with speed as did the yield at the knee and ankle. These results indicate that spatiotemporal and kinematics adjustments with speed are mainly controlled at a spinal level (i.e., spinal locomotor CPG interacting with sensory feedback from the limbs).

In all three conditions, the burst duration of extensor muscles decreased with speed but the modulation, inferred by slope values, was less in the spinal state (Figure 5 and Table 2). Although flexor burst durations varied little with speed, we did observe a significant decrease in the intact state during quadrupedal locomotion for IP, SRT, ST and BFP muscles (see also Frigon et al., 2015). The modulation was also smaller in the spinal state. Adjustments to different treadmill speeds in spinal cats are controlled by sensory feedback (Forssberg et al., 1980a,b; Smith et al., 1982; Barbeau and Rossignol, 1987; Frigon et al., 2013), whereas a role of descending inputs is likely involved in intact animals (Ryczko et al., 2017). Mean EMG amplitude generally increases in all muscles with speed in intact and spinal cats (Smith et al., 1982; Pierotti et al., 1989; Frigon et al., 2015, 2017; Harnie et al., 2019). We also observed a linear increase in mean EMG amplitude of all analyzed muscles. In contrast to burst durations, the modulation of burst amplitudes was not consistently affected by condition or state and was muscle specific (Table 2). Such changes in EMG duration and amplitude as a function of speed have been reported in other species, including humans (Grillner, 1981; Nilsson et al., 1985; Roy et al., 1991; Andersson et al., 1997; Ivanenko et al., 2006). Furthermore, although occasionally present at moderate speeds, studies have shown that the second burst of the ST muscle is more consistent when speed increased in the Intact4 condition (Chanaud et al., 1991; Smith et al., 1993). Here we show that in the Intact2 condition, the second ST muscle burst are consistently present regardless of speed (Table 4). In the spinal state, the percentage of second bursts in the ST muscle increased with speed, whereas the incidence of second bursts in the BFP muscle remained below 10%, again highlighting differential control of these close synergists for knee flexion and hip extension. The loss of the second burst in the BFP muscle could explain the overshoot at the end of swing in spinal cats, as activation of the ST and BFP muscles in late swing were proposed to decelerate forward movement of the limb (Wisleder et al., 1990; Smith et al., 1993; Pratt et al., 1996).

### Conclusions and Perspectives

In the present study, we observed greater similarity in spatiotemporal and EMG variables between the two hindlimb-only conditions. Some changes can be attributed to state (e.g., convergence in the proportion of stance and swing durations at high speed, coordination of ankle and hip joint, switch of timing in burst activations of ST and SRT muscles, modulation of burst durations with speed, incidence of second bursts) while other changes relate more to the hindlimb-only nature of the locomotion (e.g., distance of paw placement relative to the hip, knee and ankle yield, burst durations of MG and LG, onset of extensor muscles). This reinforces the idea that most spatiotemporal and EMG variables are controlled by spinal locomotor networks interacting with sensory feedback from the limbs but also that posture and forelimb movements influence the hindlimb locomotor pattern. In other words, some changes in the hindlimb pattern observed in the spinal state can be explained by the absence of forelimb movement, and not because of removing brain commands. Thus, as de Leon et al. (1998) stated in their paper, the more appropriate pre-spinal control is hindlimb-only rather than quadrupedal stepping. Our results have important implications for the design of future experiments to study the recovery of locomotion after spinal cord injury. We are currently investigating the locomotor pattern during quadrupedal locomotion in the spinal state to better characterize state- and condition-dependent changes in the locomotor pattern.

## Data Availability Statement

The raw data supporting the conclusions of this article will be made available by the authors, without undue reservation.

## Ethics Statement

The animal study was reviewed and approved by Comité Facultaire de Protection des Animaux (CFPA-FMSS).

## Author Contributions

JH: conceptualization, methodology, formal analysis, investigation, and writing – original draft. JA: formal analysis, investigation, writing review and editing. SM, CL, AM, and GG: investigation, writing review and editing. IR and BP: funding acquisition, writing review and editing. AF: conceptualization, writing – original draft, writing review and editing, supervision, and funding acquisition.

## Conflict of Interest

The authors declare that the research was conducted in the absence of any commercial or financial relationships that could be construed as a potential conflict of interest.

## Publisher’s Note

All claims expressed in this article are solely those of the authors and do not necessarily represent those of their affiliated organizations, or those of the publisher, the editors and the reviewers. Any product that may be evaluated in this article, or claim that may be made by its manufacturer, is not guaranteed or endorsed by the publisher.
